# Open Approach to the Transversus Abdominis Plane in Horses: A Cadaver Feasibility Study

**DOI:** 10.3390/vetsci11010051

**Published:** 2024-01-22

**Authors:** Maia R. Aitken, Dario A. Floriano, Klaus Hopster

**Affiliations:** 1 Department of Clinical Studies—New Bolton Center, School of Veterinary Medicine, University of Pennsylvania, Kennett Square, PA 19348, USA; 2 Department of Clinical Science and Advanced Medicine—Matthew J. Ryan Hospital, School of Veterinary Medicine, University of Pennsylvania, Philadelphia, PA 19104, USA

**Keywords:** colic surgery, abdominal surgery, open TAP, balanced/multimodal anesthesia and analgesia, loco-regional anesthesia

## Abstract

**Simple Summary:**

Peri-incisional infiltration of local anesthetic as well as ultrasound-guided nerve blocks prior to the beginning of abdominal surgery have shown to decrease both intra-and postoperative nociception in our patients, but they can be time- and cost-intensive. A more simplified technique to desensitize the body wall during surgery was investigated in this cadaver study. In this study, the number of attempts to perform the local block as well as the success rate and spread of a dye simulating the local anesthetic were evaluated. We were able to show that it had a satisfactory success rate and was easy and quick to perform. This is the first description of this new and simplified transversus abdominis plane block in horse cadavers. Additional clinical cases will have to be investigated to determine if there is an effect on recovery from anesthesia and postoperative pain.

**Abstract:**

The study’s objective was to evaluate the feasibility and dispersion of an open approach to the transversus abdominis plane (TAP) block in eight adult equine cadavers. A ventral midline incision was made, starting 2 cm cranial to the umbilicus and extending 25 cm cranially. In total, 0.5 mL/kg of new methylene blue (NMB) was injected per horse, divided into six injections. Using an 18 g, 8 cm Tuohy needle, three injections were made per side. The needle was guided blindly into the TAP using palpation. A 60 mL syringe was attached directly to the needle, depositing ~0.08 mL/kg at each site. The time to complete the injections was recorded for each cadaver. Following injection, the ventral body wall was dissected to determine if the dye was present within the TAP space as well as to measure the extent of the dispersion of the dye, the cranial to caudal extent, and the width of the dye’s spread. Complete deposition of NMB into the TAP (six of six sites) was achieved in 5/8 horses. The median time needed to perform all the injections was 263 s. Increased adiposity (retroperitoneal fat) was associated with unsuccessful injections. This approach to the TAP was easily and quickly performed, though less successful in horses with increased retroperitoneal fat and increased BCS.

## 1. Introduction

Pain and discomfort after an exploratory laparotomy in horses can contribute to postoperative complications, such as a high incidence of colic [1]. Incisional pain has both visceral and somatic components, and regional anesthesia has been shown to provide satisfactory analgesia, decrease perioperative opioid consumption, and improve the quality of recovery in many species [2,3,4,5]. Peri-incisional infiltration of local anesthetic and ultrasound-guided fascial plane blocks, such as blocks of the transversus abdominis plane (TAP) and the rectus abdominis sheath plane (RAP), prior to the beginning of surgery have shown to decrease both intra-and postoperative nociception in human and veterinary patients, and have been described in horses as a means of multimodal anesthesia [6,7,8]. Specifically, the TAP block involves the injection of a local anesthetic into the fascial plane between the transversus abdominis and either the internal oblique or the rectus abdominis muscles, where the ventral branches of the thoracolumbar spinal nerves are located [9,10]. Based on anatomical similarities observed in the innervation of the abdominal wall between species, this technique has been extrapolated to animals undergoing abdominal surgery (e.g., dogs, cats, horses, calves, lynxes, chinchillas, pigs) [11,12,13,14,15,16,17].

In horses, the lateral and ventral abdominal walls are innervated by the ventral branches of the 10th to 18th thoracic spinal nerves (T10–T18) that run within the fascial plan [18]. Several studies have been published regarding the performance of the TAP block under ultrasound guidance in horses [7,13,19]. These techniques are usually performed preoperatively and require preoperative steps, either standing or after general anesthesia has been induced. Following induction and once patients are draped, however, access to the “traditional approaches” is precluded. In cases of colic, horses with severe gastrointestinal lesions may require immediate surgical intervention. In these cases, minimizing the time to prepare the patient is critical, and such preoperative techniques are often skipped.

In human medicine, an intraoperative, surgeon-administered TAP block has been described. With this technique, the surgeon injects directly through the transversus abdominis muscle using a loss of resistance or the feeling of ‘one pop’ [20,21,22]. Recently, a similar approach has been described in cadaver dogs [23]. This approach, because it allows for direct visualization of the deposition of the injectate after the incision has been created, could be performed after surgery prior to closure of the body wall, without affecting the preoperative time and potentially prolonging the effect of the local anesthetic in the postoperative period [20,21,22]. A search in the Medline database found no studies investigating the use of an open approach to the TAP in horses.

In this study, we aimed to investigate the feasibility of an open approach to the TAP in horse cadavers undergoing ventral midline celiotomy positioned in dorsal recumbency.

## 2. Materials and Methods

A cadaver study was designed to evaluate the spread of a local anesthetic solution within the TAP of horses using an open, intra-abdominal approach. Eight fresh horse cadavers of various breeds, namely Thoroughbred (3), Quarter Horse (2), Standardbred (1), Appaloosa (1), and pony (1), weighing between 150 and 630 kg (median weight: 485 kg) that had been recently euthanized for reasons unrelated to the study were investigated.

**Figure 1:**
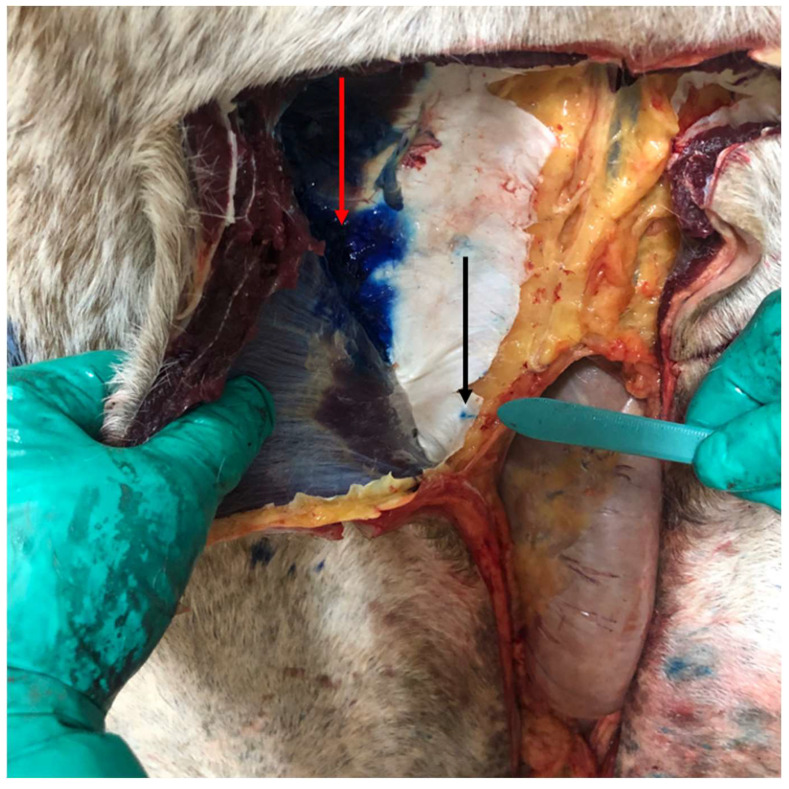
Dissection of the cadaver. Site of the needle’s insertion at the cranial edge of the incision (black arrow). Deposition of new methylene blue within the TAP lateral and cranial to the incision (red arrow).

**Figure 2:**
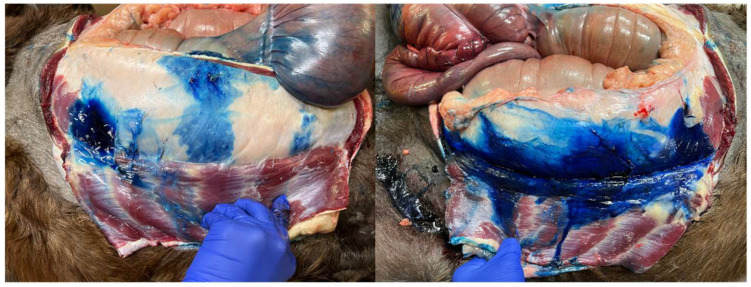
Cadaver of Horse 8, depicting four of six successful injections (two retroperitoneal). Note the substantial amount of retroperitoneal fat visible at the incisional edge.

Following euthanasia, the horses were placed in dorsal recumbency, and the ventral abdomen was clipped. A ventral midline incision was made starting 2 cm cranial to the umbilicus and extending cranially for approximately 25 cm. In the case of the pony, a 15 cm incision was made. Using a #10 scalpel, the skin was incised from approximately 2 cm cranial to the umbilicus to the desired length. Sharp dissection was continued through the subcutaneous tissue. A midline incision was made through the linea alba using the same #10 blade as if to approach a ventral midline exploratory celiotomy. Digital dissection was used to penetrate the retroperitoneal fat and peritoneum to gain access to the abdomen. In total, approximately 0.5 mL/kg of new methylene blue (NMB) was injected per horse in line with a previous study in cadaver dogs [9]. This volume was divided into six injection sites in total per horse to perform the open TAP injections. Using an 18 g, 8 cm Tuohy needle, three sites along the length of each side of the incision were injected left and right, at the cranial edge of the incision, the middle of the incision, and the caudal edge of the incision. The needle was inserted from an intraincisional approach at a site perpendicular to the cut incisional edge, or parallel to the body wall, just lateral to the *linea alba* (approximately 5 mm from the incision; see Figure 1 and Figure 2). At this site, the needle was inserted blindly through the peritoneal lining, retroperitoneal fat, and dorsal rectus sheath, using palpation and guidance of the nondominant hand. If the incisional was not perfectly midline and the edge had exposed of the rectus muscle, the needle was directly inserted between the dorsal border of the rectus muscle and the dorsal rectus sheath (i.e., between the rectus abdominis muscle and the internal or dorsal rectus sheath). Once inserted, the needle was blindly advanced laterally approximately 8 cm into the plane, guided by sensation of the needle in the fascial plane, as well as intra-abdominal palpation using the opposite hand within the abdomen. A 60 mL Luer-lock syringe was attached directly to the needle, and ~0.08 mL/kg of NMB was deposited at each site (40 mL per site for full sized horses, 12 mL for the pony). The right and left sides of the incision were performed from the contralateral side of the horse so that the right body wall was injected from the left side of the horse and vice versa. All injections were performed by the same investigator (M.R.A.).

The number of attempts to advance the needle into the TAP per injection site was recorded. The time to complete the injections was measured for each cadaver, including the time for the operator to walk from one side to the other. Following injections, the ventral body wall was dissected from the peritoneal lining and the dorsal or internal rectus sheath to determine if the injection was successful, as determined by dye being present within the TAP, as well as to measure the extent of the dispersion of the dye. The cranial to caudal spread was measured from the cranial and caudal edges of the incision, respectively, and the maximal width of dye spread on each side was also measured. If the injection was unsuccessful and dye was not within the TAP, the location of the dye was recorded.

Data were collected from all cadavers. No sample size was calculated for the research, and the number was based on the number of cadavers obtained in the time frame of the study. The distribution of the variables was tested for normality using the Shapiro–Wilk test. For normally distributed variables, the descriptive analysis of the sample was performed in terms of the mean and standard deviation (SD); for non-normally distributed variables, the descriptive analysis was performed in terms of median and range.

## 3. Results

In total, 48 injections were performed in eight equine cadavers (six injections per horse). The total number of attempts to place the needle was 63, resulting in a median number of 1.4 times per injection site (range: 1.2–1.8 attempts per site). In 41 of 48 injections, successful deposition of NMB within the TAP space was observed following examination of the dissected cadaver; in five injections, the deposition was retroperitoneal, and in two injections, deposition was intramuscular.

In 50% (4/8) of cadavers, all six injections were successful. One cadaver had successful deposition in five of six injections (one injection was retroperitoneal), whereas in the remaining three equine cadavers, four of six injections were performed successfully (see Table 1). Horses 5, 7, and 8 were noted to have significant amounts of retroperitoneal fat deposition.

The spread of dye did not differ between the right and left side of the incision. The median cranial extent of the dye’s spread was 10.5 cm on the right side and 11 cm on the left side, with a range of 0–12 cm for both sides. The median caudal extent of the dye was 7.5 cm on the right and 7 cm on the left, with a range of 2–15 cm for both sides. The median width of the dye on the right was 15.5 cm, with a range of 11–18 cm. The median width of the left dye was 15 cm, with a range of 10–20 cm. The median time for completing the six injections per horse was 263 s (range: 185–403 s), including moving from one side of the table to the other.

**Scheme 1:**
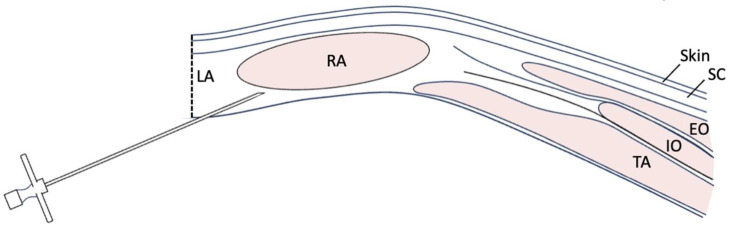
Cross-sectional schematic of the body wall with the site of the Tuohy needle’s insertion. The dotted line represents the midline of the incisional edge. The needle is then flattened out and advanced blindly into the TAP space using intra-abdominal guidance and palpation with the nondominant hand. LA—linea alba; RA—rectus abdominis; SC—subcutaneous tissue; EO—external abdominal oblique; IO—internal abdominal oblique; TA—transversus abdominis.

**Figure 3:**
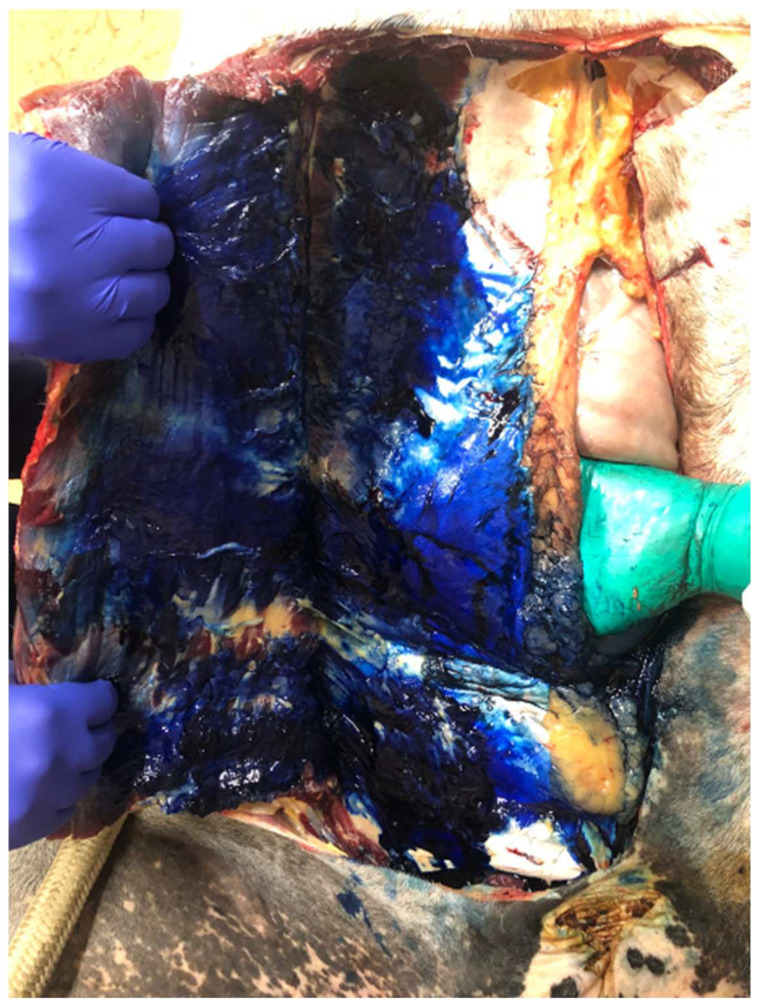
Cadaver of Horse 6, depicting dissection of the ventral abdomen and successful deposition within the TAP.

**Figure 4:**
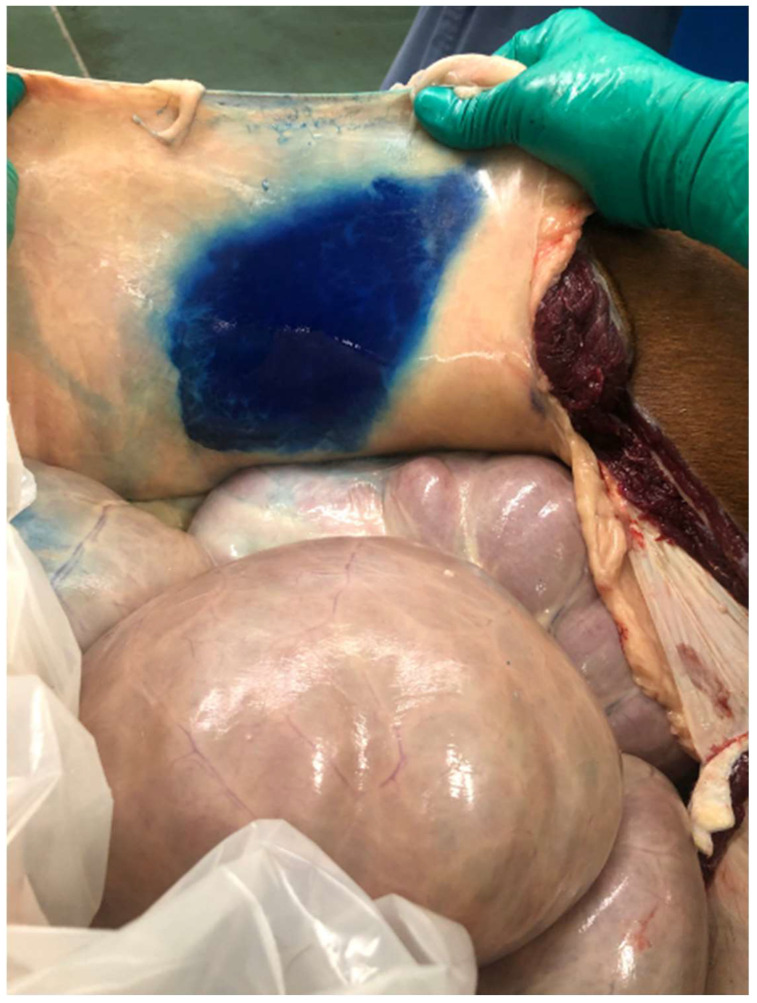
Dissection of the cadaver of Horse 1, depicting the retroperitoneal deposition of new methylene blue.

Demographic data from all eight horses and the success rate of NMB within the TAP are shown in Table 1. Images of cadaver dissection are shown in Figure 1, Figure 2, Figure 3 and Figure 4 and Scheme 1.

## 4. Discussion

This study demonstrated the feasibility of an open approach to the TAP in horse cadavers positioned in dorsal recumbency. Injections of NMB were correctly performed 85% of the time. In 50% of horse cadavers, all six injections were successful. Out of four horses with incomplete injections, three horses had large amounts of retroperitoneal fat noted. In human medicine, it is recognized that in obese patients, the performance of this block can be challenging due to excessive subcutaneous fat and the increased depth of the TAP [24,25]. 

Except for a cadaver where only four injections were successful, in all horses, the use of approximately 0.5 mL/kg divided into six injections resulted in staining of the TAP all around the surgical incision for several centimeters, in the cranial, caudal, and lateral directions. We have found no studies investigating open approaches to the TAP in horses and therefore, it is difficult to compare our findings with the current literature. 

The TAP block relies on a large volume of injectate to cover several spinal nerves. Furthermore, the spread of tissue staining depends also on the technique and the properties of the injected substance [26]. In the open TAP approach in dogs, either 1 mL/kg or 0.5 mL/kg was administered successfully through single bilateral injections using an intra-abdominal approach via a 5 cm surgical incision [23]. This injection was directly observed via the incision, a technique that cannot be duplicated in horses due to their large size and robust body wall. On the basis of previous similar studies in dogs and horses [13,19,23], we attempted a low-dose total volume injection of 0.5 mL/kg via three injections per side in the horse. Potential toxicity and the effectiveness of different concentrations of local anesthetics should be considered before implementing this technique. The volume we used would equal a 10 mg/kg total dose of 2% lidocaine in a 500 kg horse. Dilution of 2% lidocaine 1:1 with sterile saline would allow the same total volume of 0.5 mL/kg with a total amount of 5 mg/kg lidocaine. A limitation of this technique is the blind nature of the procedure, relying on tactile feedback from the penetration of the needle through the peritoneum and the dorsal rectus sheath. Additional guidance was performed by a hand palpating within the abdomen; however, this technique did not provide tactile feedback in horses with large amounts of retroperitoneal fat. Therefore, injections were less successful in these cases. Particularly in horses with a greater amount of peritoneal fat and therefore reduced palpability, the use of the Tuohy needle seemed to be helpful. Furthermore, there was a noticeable difference in resistance when injecting into the TAP versus into the muscular layers. In cases of accidental intramuscular injection, resistance to injection was felt, in addition to a noticeable and palpable bump on the skin’s surface. In contrast, retroperitoneal injection was not as easily notable and had similar resistance to the injection into the TAP. The retroperitoneal fat has “webs” of connective tissue that can mimic the sensation of penetrating through the dorsal rectus sheath. Injection in this area was performed without appreciable resistance.

In other equine studies where the needle was advanced through the skin under ultrasound guidance, injections were made further dorsolateral from the linea alba, with one or more injections between the last rib and the iliac crest, and the volumes of injectates used were between 0.2 and 1 mL/kg [7,19]. Because, in horses, the lateral and ventral abdominal wall are innervated by the ventral branches of 10th to 18th thoracic spinal nerves, these cadaveric studies evaluated the staining of the nerves after dissection, showing that even with 0.5 mL/kg, a single injection provided limited staining, and two or three injections may be needed in order to stain all the relevant nerves. Nerves were considered to be positively stained when ≥1 cm of the length was stained [7,13,19]. In our study, we did not look specifically at the staining of single nerves but measured the spread of the contrast from the incision instead. Except for one horse, the TAP around the surgical incision was stained in all horses for far more than 1 cm, suggesting that the nerves in that area were positively stained according to those criteria. 

The limitations of this study include the fact that we only used cadavers, and the subsequent anti-nociceptive effect of this approach is yet unknown. Nevertheless, the dispersion of the local anesthetic solution within the fascial planes of cadavers is expected to differ from that in living animals. The fascial planes exhibit dynamic properties and the transmission of muscle movements to the fascia facilitates the transport of local anesthetic within the fascial plane. Consequently, it is expected that the local anesthetic solution will spread more extensively from the injection site in living animals compared with cadavers [27]. In addition, this approach requires the deposition of local anesthetic after the initial surgical approach is performed. Such a block would likely be performed at the end of the exploration of the abdomen in a colic surgery and before incisional closure. Thus, this approach would have no effect on the MAC requirement during the majority of the surgical procedure. Instead, this approach may be useful for the reduction in MAC during incisional closure and may have an effect on the quality of recovery from anesthesia. However, this would need to be assessed in a separate study with live horses and/or clinical cases.

The open approach to the TAP in horse cadavers performed in our study was easy to perform and required between 3 and 6.5 min to be completed. Although this method would not provide any additional analgesia at the time of incision, the preoperative time would not be affected. Additionally, performing this technique following correction of the lesion may facilitate incisional closure by decreasing the patient’s discomfort, thereby by decreasing tension or rolling of the body wall and the subsequent time of incisional suturing. 

Further, using long-acting local anesthetics could extend the analgesic effect in the postoperative period, potentially decreasing the need for systemic analgesics and the associated side effects. Additional studies are necessary to assess whether this approach would have any effect on the reduction in MAC or quality of recovery from anesthesia. 

## 5. Conclusions

In this cadaver study, the described open TAP technique was relatively quick and easy to perform, and had a good success rate. Clinical cases will have to be investigated to determine if there is a repeatable effect on body wall tone, recovery from anesthesia, and pain in the postoperative period.

## Figures and Tables

**Table 1:** Demographic data and degree of spread of the dye from eight equine cadavers.

Individuals	Weight (kg)	BCS	Volume Injected per Site (mL)	Cranial Spread to the Right (cm)	Cranial Spread to the Left (cm)	Caudal Spread to the Right (cm)	Caudal Spread to the Left (cm)	Width of the Dye’s Spread on the Right (cm)	Width of the Dye’s Spread on the Left (cm)	Success Rate
Horse 1	480	4	40	12	10	6	6	18	16	83% 1 RP
Horse 2	150	6	12	6	7	5	6	11	10	100%
Horse 3	490	5	40	10	11	7	6	16	13	100%
Horse 4	470	4	40	11	12	12	13	17	15	100%
Horse 5	560	6	40	11	11	2	4	15	17	67% 1 RP, 1 IM
Horse 6	440	4	40	12	11	15	14	15	15	100%
Horse 7	630	8	40	8	12	8	15	17	20	67% 1 RP, 1 IM
Horse 8	610	7	40	0	0	8	8	15	15	67% 2 RP

IM, Intramuscular; RP, Retroperitoneal.

## Data Availability

All data supporting the reported results are contained within the article.
